# The decline of radical nephrectomy: Contemporary trends in the treatment of T1 renal cell carcinoma

**DOI:** 10.1002/bco2.70148

**Published:** 2026-01-04

**Authors:** Zorawar Singh, Dylan Brown, Justin James, Atieh D. Ashkezari, Manish A. Vira, Arun Rai

**Affiliations:** ^1^ Department of Urology Northwell Health New Hyde Park New York USA; ^2^ City University of New York School of Medicine New York New York USA; ^3^ Department of Urology James Buchanan Brady Urological Institute at Johns Hopkins Hospital Baltimore Maryland USA

**Keywords:** ablative therapy, nephron‐sparing surgery, partial nephrectomy, renal cell carcinoma, SEER database, treatment trends

## Abstract

**Introduction:**

The diagnosis of small renal masses is becoming increasingly common. Management recommendations are shifting from radical nephrectomy (RN) toward nephron‐sparing options such as partial nephrectomy (PN), thermal ablation (TA), and active surveillance (AS). This study aims to present current treatment trends in the USA for treating clinical stage T1 renal cell carcinoma in the largest series to date. Additionally, we sought to identify predictors linked to the receipt of ablative treatments.

**Methods:**

We conducted a retrospective cohort study using the National Cancer Institute's Surveillance, Epidemiology, and End Results (SEER) 22 Registries from 2000 to 2021. Adults (≥18 years) with unilateral, primary cT1 renal cortical renal cell carcinoma were included. Treatments analysed were RN, PN, and ablative therapies (radiofrequency, cryoablation, and laser). Annual trends were assessed, and multinomial logistic regression identified demographic and clinical predictors of treatment selection.

**Results:**

A total of 86 642 patients were included. Between 2000 and 2021, PN increased from 16% to 57% and ablation from 1% to 11%, while RN decreased from 84% to 33% (*p* < 0.001). Overall, 6138 ablative treatments were performed, with the majority (*n* = 5623, 92%) conducted on renal masses <4 cm. The most substantial growth was for tumours <2 cm, with a 17.2% increase in ablation compared to a 3.4% increase for masses >4 cm. Among ablative techniques, cryoablation's utilization increased most dramatically from 0% to 7% during the study period. Multivariable analysis indicated that predictors for choosing ablation over RN included older age, later year of diagnosis, smaller tumours, and higher income. Conversely, Hispanic ethnicity, marital status, and non‐classic RCC subtypes were linked to a higher likelihood of receiving RN versus ablation.

**Conclusion:**

Over the past two decades, PN and ablation have increasingly replaced RN in the management of cT1 renal masses, particularly for tumours <4 cm. As technology advances, ablation is likely to expand further, reinforcing the shift toward nephron‐sparing strategies.

## 1 INTRODUCTION

Small renal masses are diagnosed with increasing frequency.^1^ Recommended management of these masses has changed over time, with a trend away from radical nephrectomy (RN) toward less invasive approaches including partial nephrectomy (PN), thermal ablation (TA), and active surveillance (AS).^2^, ^3^

The shift from RN to PN has been driven by observational^4^ and experimental^5^ data showing long‐term oncologic equivalence between RN and PN and lower risk of new‐onset chronic kidney disease (CKD) after treatment with PN. Some studies have suggested that PN even confers a long‐term survival benefit,^6^ but more recent data show a more mixed picture.^7^, ^8^ As of 2021, the AUA recommends partial nephrectomy (PN) for management of all cT1a and select cT1b tumours; RN is recommended only for patients without other renal comorbidities who are diagnosed with complex tumours that make PN less technically feasible.^9^

Percutaneous thermal ablation, either as cryoablation, radiofrequency ablation, or laser ablation, is a newer treatment modality for small, localized renal masses. Currently, TA is recommended as an alternate treatment approach for masses less than 3 cm in size.^9^ Long‐term oncological outcomes are comparable between PN and TA,^4^, ^10^ especially for smaller tumours,^10^, ^11^ with fewer perioperative complications,^4^, ^11^, ^12^, ^13^ though rates of local tumour recurrence are higher.^4^, ^13^

For select patients with small masses where the risk of intervention outweighs the benefits, active surveillance is often preferred.^14^, ^15^, ^16^, ^17^ Indolent or slow‐growing tumours can be safely monitored in appropriate patients,^15^ with more definitive treatment offered later if appropriate.

Previous surveys showed increasing adoption of PN, TA, and AS in the management of small renal masses.^2^, ^3^ Here, we use data from the SEER registry to examine the extent to which this trend has continued among clinicians in the United States from 2000 to 2021 in the largest contemporary series to date.

## 2 METHODS

A retrospective, observational cohort study was conducted utilizing the National Cancer Institute's Surveillance, Epidemiology and End Results (SEER) 22 Registries from 2000 to 2021. Our inclusion criteria included adults (≥18 years old) with unilateral, primary.

T1 renal cortical tumours (defined by SEER as ≤7 cm in greatest dimension, ICD‐10 C64.9) with diagnosed renal cell carcinoma (International Classification of Childhood Cancer: VIb Renal carcinomas) without major vein involvement, who underwent either surgical or ablative therapies.

SEER assigns a composite T‐stage variable that integrates both clinical and pathological staging information; therefore, it is not possible to distinguish cT1 from pT1 tumours within this dataset. Accordingly, all references to T1 in this study represent the SEER composite stage rather than a purely clinical or purely pathologic designation.

Surgical therapies included radical nephrectomy (RN, SEER Code A50) or partial nephrectomy (PN, SEER Code A30). Ablative therapies included radiofrequency (SEER Code A15), cryoablation (SEER Code A13 and A23), and laser (SEER Code A14 and A24).

Patients with missing or unknown data for marital status, race/ethnicity, geography, and income were excluded. RCC subtypes were split between common (clear cell carcinoma) and rare (papillary, chromophobe, etc.). Table S1 lists the ICD codes utilized to stratify common and rare subtypes of RCC.

Patients' age, sex, race/ethnicity, marital status, geography, income, year of diagnosis, histology, and tumour size were extracted from the data set. Descriptive statistics of categorical variables were reported as frequencies and proportions and analysed using Pearson's Chi‐Square. Multinominal logistic regression analysis was conducted to identify predictive factors associated with different interventions compared to RN. Variables in the analysis include age, sex, race/ethnicity, year of diagnosis, marital status, income, geography, tumour size, and histology. All statistical tests were performed using IBM SPSS v27.0 (IBM, Armonk, NY) using a two‐tailed alpha of 0.05 to denote significance.

## 3 RESULTS

**FIGURE 1:**
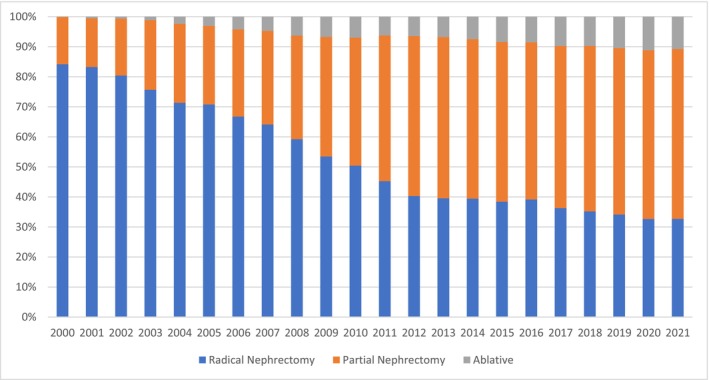
Trends in Treatment Modality Use for cT1 Renal Cell Carcinoma, 2000–2021. Annual utilization rates of radical nephrectomy, partial nephrectomy, and ablative therapies among patients with cT1 renal masses. Partial nephrectomy and ablation increased substantially over time, while radical nephrectomy declined.

**FIGURE 2:**
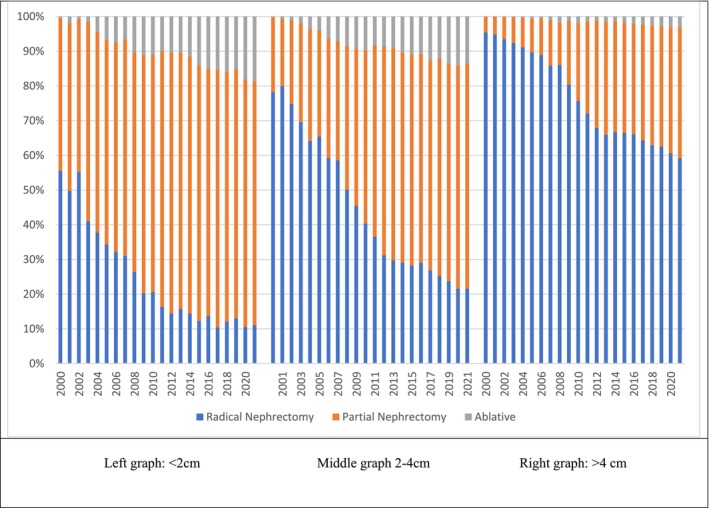
Treatment Selection by Tumour Size Category (<2 cm, 2–4 cm, and >4 cm). Proportional use of partial nephrectomy, radical nephrectomy, and ablative therapies stratified by tumour size. Nephron‐sparing approaches were most common for tumours <2 cm, whereas radical nephrectomy remained predominant for tumours >4 cm.

A total of 86 642 patients with primary cT1 renal masses diagnosed between 2000 and 2021 were included in this analysis (Table 1). We found a significant shift from radical nephrectomy toward nephron‐sparing treatment modalities (Figure 1). From 2000 to 2021 the proportion of patients treated with radical nephrectomy decreased from 84% to 33% (Table 2). Over the same time period, there was a concomitant rise in the utilization of partial nephrectomy and ablative therapies going from 16% to 57% and 0% to 11%, respectively (Table 1). This trend was most evident in patients with smaller tumours. For patients with tumours <2 cm, 55% were treated with RN in 2000, which declined to 12% by 2021 (Figure 2). Conversely, use of PN and ablation to treat these tumours increased from 16% to 57% and from 0% to 11%, respectively. RN remained the most common treatment for tumours >4 cm throughout the study period, though the use of PN and even ablation for larger cancers has increased.

**TABLE 1:** Demographic characteristics.

	PN	RN	Ablative total	Cryoablation	Radiofrequency	Laser	Total	Statistic
**Age**								<0.001
<50	10 417 (27%)	8127 (20%)	531 (9%)	346 (9%)	160 (10%)	25 (7%)	19 075 (22%)	
50–59	10 744 (28%)	10 676 (26%)	1086 (18%)	730 (18%)	282 (17%)	74 (18%)	22 506 (26%)	
60–69	11 190 (29%)	11 760 (29%)	1826 (30%)	1224 (31%)	465 (28%)	137 (34%)	24 776 (29%)	
70–79	5610 (15%)	8333 (20%)	1858 (31%)	1218 (31%)	520 (31%)	120 (30%)	15 801 (19%)	
≥80	975 (3%)	2672 (7%)	837 (14%)	527 (13%)	253 (16%)	57 (14%)	4484 (6%)	
**Sex**								0.006
Female	15 039 (39%)	16 511 (40%)	2385 (39%)	1572 (39%)	659 (40%)	154 (38%)	33 935 (40%)	
Male	23 897 (62%)	25 057 (61%)	3753 (62%)	2473 (62%)	1021 (61%)	259 (63%)	52 707 (61%)	
**Race/Ethnicity**								<0.001
NH White	28 010 (72%)	29 907 (72%)	4496 (74%)	3013 (75%)	1183 (71%)	300 (73%)	62 413 (72%)	
NH American Indian Alaskan Native	290 (1%)	382 (1%)	53 (1%)	40 (1%)	9 (1%)	4 (1%)	725 (1%)	
NH Black	3806 (10%)	4334 (11%)	626 (11%)	349 (9%)	229 (14%)	48 (12%)	8766 (11%)	
Hispanic	6830 (18%)	6945 (17%)	963 (16%)	643 (16%)	259 (16%)	61 (15%)	14 738 (17%)	
**Marital Status**								<0.001
Not Married	13 152 (34%)	15 111 (37%)	2418 (40%)	1570 (39%)	699 (42%)	149 (37%)	30 681 (36%)	
Married	25 784 (67%)	26 457 (64%)	3720 (61%)	2475 (62%)	981 (59%)	264 (64%)	55 961 (65%)	
**Geography**								<0.001
Rural	1753 (5%)	2466 (6%)	325 (6%)	242 (6%)	62 (4%)	21 (6%)	4544 (6%)	
Near metropolitan	2519 (7%)	3592 (9%)	430 (7%)	285 (7%)	110 (7%)	35 (9%)	6541 (8%)	
Metropolitan	34 664 (89%)	35 510 (86%)	5383 (88%)	3518 (87%)	1508 (90%)	357 (87%)	75 557 (88%)	
**Income**								
Less 45 k	1178 (3%)	1707 (5%)	193 (4%)	146 (4%)	32 (2%)	15 (4%)	3078 (4%)	
45 thru 60 k	4618 (12%)	6285 (16%)	848 (14%)	557 (14%)	224 (14%)	67 (17%)	11 751 (14%)	
60 k thru 75 k	11 369 (30%)	13 310 (32%)	1681 (28%)	1024 (26%)	542 (33%)	115 (28%)	26 360 (31%)	
Greater than 75 k	21 771 (56%)	20 266 (49%)	3416 (56%)	2318 (58%)	882 (53%)	216 (53%)	45 453 (53%)	
**Year of Diagnosis**								<0.001
2000	294 (16%)	1574 (84%)	2 (0%)	2 (0%)	0 (0%)	0 (0%)	1870 (100%)	
2001	353 (16%)	1814 (83%)	12 (1%)	8 (0%)	0 (0%)	4 (0%)	2179 (100%)	
2002	441 (19%)	1872 (80%)	15 (1%)	10 (0%)	0 (0%)	5 (0%)	2328 (100%)	
2003	557 (23%)	1819 (76%)	28 (1%)	16 (1%)	5 (0%)	7 (0%)	2404 (100%)	
2004	704 (26%)	1912 (71%)	64 (2%)	43 (2%)	17 (1%)	4 (0%)	2680 (100%)	
2005	742 (26%)	2006 (71%)	86 (3%)	56 (2%)	22 (1%)	8 (0%)	2834 (100%)	
2006	905 (29%)	2081 (67%)	132 (4%)	81 (3%)	32 (1%)	19 (1%)	3118 (100%)	
2007	1038 (31%)	2134 (64%)	157 (5%)	106 (3%)	36 (1%)	15 (0%)	3329 (100%)	
2008	1257 (34%)	2160 (59%)	229 (6%)	150 (4%)	57 (2%)	22 (1%)	3646 (100%)	
2009	1555 (40%)	2089 (53%)	264 (7%)	181 (5%)	65 (2%)	18 (0%)	3908 (100%)	
2010	1543 (43%)	1825 (50%)	252 (7%)	176 (5%)	64 (2%)	12 (0%)	3620 (100%)	
2011	1833 (49%)	1710 (45%)	236 (6%)	151 (4%)	66 (2%)	19 (1%)	3779 (100%)	
2012	2150 (53%)	1628 (40%)	261 (6%)	193 (5%)	50 (1%)	18 (0%)	4039 (100%)	
2013	2231 (54%)	1642 (40%)	281 (7%)	192 (5%)	68 (2%)	21 (1%)	4154 (100%)	
2014	2396 (53%)	1779 (39%)	338 (7%)	219 (5%)	104 (2%)	15 (0%)	4513 (100%)	
2015	2605 (53%)	1883 (38%)	413 (8%)	271 (6%)	118 (2%)	24 (0%)	4901 (100%)	
2016	2619 (52%)	1959 (39%)	426 (9%)	259 (5%)	147 (3%)	20 (0%)	5004 (100%)	
2017	2919 (54%)	1962 (36%)	528 (10%)	355 (7%)	144 (3%)	29 (1%)	5409 (100%)	
2018	3076 (55%)	1965 (35%)	543 (10%)	370 (7%)	139 (2%)	34 (1%)	5584 (100%)	
2019	3340 (55%)	2056 (34%)	631 (10%)	425 (7%)	168 (3%)	38 (1%)	6027 (100%)	
2020	2975 (56%)	1730 (33%)	592 (11%)	374 (7%)	197 (4%)	21 (0%)	5297 (100%)	
2021	3403 (57%)	1968 (33%)	648 (11%)	407 (7%)	181 (3%)	60 (1%)	6019 (100%)	
**Histology Type**								<0.001
RCC Common	38 070 (98%)	40 280 (97%)	6066 (99%)	3991 (99%)	1666 (100%)	409 (99%)	84 416 (98%)	
RCC Rare	866 (3%)	1288 (4%)	72 (2%)	54 (2%)	14 (1%)	4 (1%)	2226 (3%)	
**Tumour Size**								<0.001
<=2 cm	10 174 (27%)	2901 (7%)	1764 (29%)	1155 (29%)	492 (30%)	117 (29%)	14 839 (18%)	
2‐4 cm	21 180 (55%)	15 432 (38%)	3859 (63%)	2566 (64%)	1042 (62%)	251 (61%)	40 471 (47%)	
4 cm	7582 (20%)	23 235 (56%)	515 (9%)	324 (8%)	146 (9%)	45 (11%)	31 332 (37%)	

**TABLE 2:** Comparison of Demographic, Clinical, Tumour, and Diagnosis‐Year Characteristics Across Treatment Modalities.

	PN	RN	Ablative Total	Statistic
**Age**				<0.001
<50	10 417 (27%)	8127 (20%)	531 (9%)	
50–59	10 744 (28%)	10 676 (26%)	1086 (18%)	
60–69	11 190 (29%)	11 760 (29%)	1826 (30%)	
70–79	5610 (15%)	8333 (20%)	1858 (31%)	
≥80	975 (3%)	2672 (7%)	837 (14%)	
**Sex**				0.006
Female	15 039 (39%)	16 511 (40%)	2385 (39%)	
Male	23 897 (62%)	25 057 (61%)	3753 (62%)	
**Race/Ethnicity**				<0.001
NH White	28 010 (72%)	29 907 (72%)	4496 (74%)	
NH American Indian Alaskan Native	290 (1%)	382 (1%)	53 (1%)	
NH Black	3806 (10%)	4334 (11%)	626 (11%)	
Hispanic	6830 (18%)	6945 (17%)	963 (16%)	
**Marital Status**				<0.001
Not Married	13 152 (34%)	15 111 (37%)	2418 (40%)	
Married	25 784 (67%)	26 457 (64%)	3720 (61%)	
**Geography**				<0.001
Rural	1753 (5%)	2466 (6%)	325 (6%)	
Near metropolitan	2519 (7%)	3592 (9%)	430 (7%)	
Metropolitan	34 664 (89%)	35 510 (86%)	5383 (88%)	
**Income**				
Less 45 k	1178 (3%)	1707 (5%)	193 (4%)	
45 thru 60 k	4618 (12%)	6285 (16%)	848 (14%)	
60 k thru 75 k	11 369 (30%)	13 310 (32%)	1681 (28%)	
Greater than 75 k	21 771 (56%)	20 266 (49%)	3416 (56%)	
**Histology Type**				<0.001
RCC Common	38 070 (98%)	40 280 (97%)	6066 (99%)	
RCC Rare	866 (3%)	1288 (4%)	72 (2%)	
**Tumour Size**				<0.001
<=2 cm	10 174 (27%)	2901 (7%)	1764 (29%)	
2‐4 cm	21 180 (55%)	15 432 (38%)	3859 (63%)	
4 cm	7582 (20%)	23 235 (56%)	515 (9%)	
**Year of Diagnosis**				<0.001
2000	294 (16%)	1574 (84%)	2 (0%)	
2001	353 (16%)	1814 (83%)	12 (1%)	
2002	441 (19%)	1872 (80%)	15 (1%)	
2003	557 (23%)	1819 (76%)	28 (1%)	
2004	704 (26%)	1912 (71%)	64 (2%)	
2005	742 (26%)	2006 (71%)	86 (3%)	
2006	905 (29%)	2081 (67%)	132 (4%)	
2007	1038 (31%)	2134 (64%)	157 (5%)	
2008	1257 (34%)	2160 (59%)	229 (6%)	
2009	1555 (40%)	2089 (53%)	264 (7%)	
2010	1543 (43%)	1825 (50%)	252 (7%)	
2011	1833 (49%)	1710 (45%)	236 (6%)	
2012	2150 (53%)	1628 (40%)	261 (6%)	
2013	2231 (54%)	1642 (40%)	281 (7%)	
2014	2396 (53%)	1779 (39%)	338 (7%)	
2015	2605 (53%)	1883 (38%)	413 (8%)	
2016	2619 (52%)	1959 (39%)	426 (9%)	
2017	2919 (54%)	1962 (36%)	528 (10%)	
2018	3076 (55%)	1965 (35%)	543 (10%)	
2019	3340 (55%)	2056 (34%)	631 (10%)	
2020	2975 (56%)	1730 (33%)	592 (11%)	
2021	3403 (57%)	1968 (33%)	648 (11%)	

The growth in ablation treatment is primarily driven by the use of ablation to treat smaller masses: the majority of ablations (*n* = 5623, 92%) were used for masses <2 cm. From 2000 to 2021, ablation for these masses increased 17.2%, compared to a 3.4% increase for masses <4 cm. The most common ablation treatment utilized was cryoablation followed by radiofrequency and then laser ablation. Multivariable analysis revealed several predictors for the choice of ablation therapy over more invasive modalities. These predictors included older age, a later year of diagnosis, smaller tumors, and higher income. Conversely, patients who were married, of Hispanic ethnicity, and who were diagnosed with rare histological subtypes of RCC were less likely to receive ablation therapy (Table 3).

**TABLE 3:** Multinominal Logistic Regression, Reference: Radical Nephrectomy.

	Partial Nephrectomy	*p*‐value	Ablative Therapy	*p*‐value
	OR (95% CI)		OR (95% CI)	
**Age (Ref: <50)**				
50–59	0.79 (0.75–0.82)	<0.001	1.59 (1.43–1.78)	<0.001
60–69	0.73 (0.7–0.76)	<0.001	2.41 (2.17–2.68)	<0.001
70–79	0.56 (0.53–0.59)	<0.001	3.86 (3.46–4.29)	<0.001
≥80	0.39 (0.36–0.43)	<0.001	7.62 (6.69–8.67)	<0.001
**Sex (Ref: Female)**				
**Year of Diagnosis**	1.12 (1.12–1.13)	<0.001	1.17 (1.16–1.17)	<0.001
**Race (Ref: NH White)**				
NH American Indian Alaskan Native	0.71 (0.6–0.85)	<0.001	0.93 (0.68–1.27)	0.638
NH Black	0.86 (0.81–0.9)	<0.001	0.97 (0.88–1.07)	0.489
Hispanic	0.83 (0.79–0.87)	<0.001	0.89 (0.82–0.97)	0.006
**Marital Status (Ref: Not Married)**				
Married	1.12 (1.08–1.16)	<0.001	0.86 (0.81–0.91)	<0.001
**Income (Ref: Less 45 k)**				
45 thru 60 k	1.05 (0.95–1.16)	0.368	1.20 (0.99–1.44)	0.06
60 k thru 75 k	1.31 (1.18–1.45)	<0.001	1.26 (1.04–1.52)	0.018
Greater than 75 k	1.42 (1.28–1.58)	<0.001	1.39 (1.15–1.68)	<0.001
**Geography (Ref: Rural)**				
Near metropolitan	0.96 (0.88–1.05)	0.397	0.87 (0.74–1.03)	0.098
Metropolitan	1.14 (1.05–1.24)	0.002	0.96 (0.83–1.11)	0.578
**Tumour Size (Ref: <=2 cm)**				
2‐4 cm	0.38 (0.36–0.4)	<0.001	0.32 (0.3–0.35)	<0.001
4 cm	0.08 (0.08–0.09)	<0.001	0.03 (0.02–0.03)	<0.001
**Histology (Ref: RCC Common)**				
RCC Rare	0.76 (0.68–0.84)	<0.001	0.36 (0.28–0.46)	<0.001

## 4 DISCUSSION

The treatment modalities used to manage small renal malignancies have continued to evolve over the past two decades. Our data demonstrate that the previously documented trend away from RN and toward less invasive, nephron‐sparing modalities such as PN and ablation has persisted throughout the study period.^2^, ^3^ While RN was the predominant approach in 2000, it is now largely reserved for masses >4 cm. PN has become the standard for cT1 renal tumours, while ablation has gained increasing utilization, particularly for smaller lesions. A major contributor to the increasing utilization of partial nephrectomy has been the widespread adoption of robotic‐assisted surgery beginning in the mid‐2000s.^18^ Robotics reduced the technical complexity of PN, facilitated tumour excision, and broadened surgeon adoption nationwide. Although SEER does not reliably code robotic procedures across all study years, preventing direct quantification, national robotic trends closely mirror the rise of PN observed in our dataset.

The shift toward PN and ablation is likely driven by the growing consensus that these nephron‐sparing techniques achieve oncologic outcomes comparable to RN while minimizing declines in renal function^4^, ^16^ and overall morbidity. RN, though historically the mainstay and still associated with excellent oncologic control, carries a greater risk of post‐operative CKD and reduced overall survival related to diminished renal reserve.^4^, ^16^ PN and ablation, by contrast, preserve renal parenchyma and are associated with improved long‐term renal function, which has become an increasingly important consideration given the morbidity associated with CKD. Given the dangers of CKD, clinicians have increasingly favoured nephron‐sparing techniques over RN for all but the most complex and advanced tumours.

As management options for small renal tumours continue to advance, several emerging technologies have the potential to further improve nephron‐sparing treatments and increase their utilization. Three‐dimensional and holographic imaging are improving preoperative planning by allowing precise evaluation of tumour anatomy and vasculature.^19^, ^20^ Augmented reality platforms are being integrated into robotic consoles, enhancing intraoperative visualization and potentially reducing operative complexity.^21^ Fluorescence imaging has been employed to assess real‐time perfusion, potentially minimizing ischemic injury to normal renal tissue and improving post‐operative outcomes.^22^ These developments parallel improvements enabled by robotic surgery, reinforcing how visualization‐enhancing technologies continue to expand the feasibility of nephron‐sparing approaches.^23^

Similarly, advances in ablative therapies could expand their applicability. Currently, ablation is recommended only for tumours less than 3 cm in size,^9^ based largely on studies demonstrating a decrease in oncologic efficacy for renal tumours >3 cm.^24^ However, emerging ablative technologies may eventually change this paradigm. Histotripsy, in which targeted ultrasound waves are used to induce mechanical breakdown of tumour tissue, has shown promising outcomes in treating liver tumours and is currently being investigated for treating kidney tumours as well.^25^, ^26^ Histotripsy has several advantages over standard ablation methods: it is non‐invasive, extremely precise, and allows real‐time visualization of the ablation.^27^ Should comparable outcomes be demonstrated in RCC, histotripsy may accelerate the integration of ablation into the treatment paradigm for larger and more complex lesions. Other emerging modalities in renal ablation include irreversible electroporation.^28^ While initial studies demonstrated acceptable safety profiles, oncologic efficacy has been modest, and further investigations are ongoing.^28^

Another important factor influencing contemporary treatment patterns is the expanding role of active surveillance (AS) for small renal masses. SEER does not differentiate AS from watchful waiting and does not reliably capture patients managed without intervention, resulting in their exclusion from our analytic cohort. Updated AS outcomes from large prospective registries demonstrate excellent cancer‐specific survival and low metastatic progression in appropriately selected cT1a tumours.^29^ Increasing AS adoption may shift very small or indolent tumours away from intervention altogether, indirectly influencing the observed rates of PN and ablation.

Several limitations merit consideration. First, the retrospective design of this SEER‐based study introduces potential selection bias and precludes causal inference. Second, due to the limited granularity of the SEER database, we were unable to distinguish between patients managed with active surveillance versus watchful waiting. As a result, these important management strategies for small renal masses were excluded from analysis and the focus was on interventional treatments. Third, misclassification bias is possible given reliance on coding for treatment type and histology. Additionally, substantial provider‐ and institution‐level variation influences treatment selection.^30^ Surgeon experience, institutional preference, and access to robotics or ablation technology can significantly affect management decisions and are not captured in SEER, contributing to treatment variability beyond patient and tumour characteristics. Finally, although SEER provides a large and diverse population‐based sample, its findings may not fully capture treatment patterns in non‐SEER regions or reflect practices in other countries.

## 5 CONCLUSION

In the largest contemporary study to date including 86 642 patients with cT1 RCC, we observed a significant decline in the use of RN and a marked increase in PN and ablative therapies over two decades. Predictors of ablation included older age, later year of diagnosis, smaller tumour size, and higher income, whereas Hispanic ethnicity, marital status, and rare histologic subtypes were associated with continued RN use. These findings highlight the ongoing shift toward nephron‐sparing strategies in the management of small renal masses. As novel technologies such as advanced imaging and histotripsy mature, the role of ablation is likely to expand further, reinforcing the movement away from radical nephrectomy in favour of personalized, kidney‐preserving approaches.

## AUTHOR CONTRIBUTIONS

Zorawar Singh contributed to the conception and design of the study, data acquisition, data analysis and interpretation, and drafting of the manuscript. Dylan Brown contributed to drafting of the manuscript, data analysis, and critical revision of the manuscript. Justin James contributed to data acquisition, data interpretation, data analysis, and drafting of the manuscript. Atieh Ashkerazi contributed to data acquisition and critical review of the manuscript. Manish Vira contributed to study design, supervision of the research, and critical revision of the manuscript. Arun Rai contributed to study conception and design, oversight of data interpretation, and final approval of the manuscript.

## CONFLICTS OF INTEREST

The authors declare no conflicts of interest.

## Supporting information


**Data S1.** Supporting Information.
