# Supplementary material for: The decline of radical nephrectomy: Contemporary trends in the treatment of T1 renal cell carcinoma
Source: BJUI Compass. 2026 Jan 4;7(1):e70148. doi: 10.1002/bco2.70148 (PMC12765420; doi:10.1002/bco2.70148)
Supplement: Supplementary file 1 — Data S1. Supporting Information. [file BCO2-7-e70148-s001.docx]

**Supplementary Information**

Common included ICD 3: 8312/3

Rare included ICD 3: 8050/3, 8140/3, 8211/3, 8230/3, 8263/3, 8290/3, 8311/3, 8316/3, 8318/3, 8319/3, 8320/3, 8323/3, 8480/3, 8481/3, 8504/3, 8510/3, 8550/3, 8317/3, 8260/3, 8310/3
